# Analysis of the reporting of adverse drug reactions in children and adolescents in Germany in the time period from 2000 to 2019

**DOI:** 10.1371/journal.pone.0247446

**Published:** 2021-03-03

**Authors:** Sarah Leitzen, Diana Dubrall, Irmgard Toni, Julia Stingl, Maike Schulz, Matthias Schmid, Antje Neubert, Bernhardt Sachs

**Affiliations:** 1 Research Division, Federal Institute for Drugs and Medical Devices, Bonn, Germany; 2 Department of Physics, Chemistry and Pharmacy, University of Southern Denmark, Odense, Denmark; 3 Institute for Medical Biometry, Informatics and Epidemiology, University Hospital of Bonn, Bonn, Germany; 4 Department of Pediatrics and Adolescent Medicine, University Hospital Erlangen, Erlangen, Germany; 5 Institute of Clinical Pharmacology, University Hospital of the RWTH Aachen, Aachen, Germany; 6 Central Research Institute of Ambulatory Health Care in Germany, Berlin, Germany; 7 Department for Dermatology and Allergy, University Hospital Aachen, Aachen, Germany; University of Sydney, AUSTRALIA

## Abstract

The objective of this study was to analyse reports on adverse drug reactions (ADRs) from Germany in the particularly vulnerable patient group of children and adolescents. Reporting characteristics, demographic parameters and off-label use were examined among others. The ratio of ADR reports per number of German inhabitants and the ratio of ADR reports per number of German inhabitants exposed to drugs were calculated and compared. These parameters were examined to derive trends in reporting of ADRs. 20,854 spontaneous ADR reports for the age group 0–17 years were identified in the European ADR database EudraVigilance for the time period 01.01.2000–28.02.2019 and analysed with regard to the aforementioned criteria. 86.5% (18,036/20,854) of the ADR reports originated from Healthcare Professionals and 12.2% (2,546/20,854) from non-Healthcare Professionals. 74.4% (15,522/20,854) of the ADR reports were classified as serious. The proportion of ADR reports per age group was 11.8% (0–1 month), 11.0% (2 months—1 year), 7.4% (2–3 years), 9.3% (4–6 years), 25.8% (7–12 years), and 34.8% (13–17 years) years, respectively. Male sex slightly dominated (51.2% vs. 44.8% females). Only 3.5% of the ADR reports reported off-label use. The annual number of ADR reports increased since 2000, even if set in context with the number of inhabitants and assumed drug-exposed inhabitants. The pediatric population declined in the study period which argues against its prominent role for the increase in the total number of ADR reports. Instead, among others, changes in reporting obligations may apply. The high proportion of serious ADR reports underlines the importance of pediatric drug safety.

## Introduction

Adverse drug reactions (ADRs) in children negatively impact on their quality of life, although no specific instrument is available to measure the negative effect [1].

In Germany, 1.7% of children taking medication on an *outpatient* basis experience at least one ADR [2]. According to systematic reviews the incidence of ADRs leading to hospital admission for children ranges from 0.4% to 10.3% (pooled estimate of 2.9% (2.6%, 3.1%)) [3]. For Germany, the respective figure was 1% [4]. In contrast, the ADR incidence in *hospitalized* children is estimated to be higher ranging from 9.53% [5] to 13.1% [6]. In comparison the incidence of hospitalisation of *adult* outpatients in Germany is 3.2% [7].

The objective of the present study was to analyse the annual number and the characteristics of ADR reports for children and adolescents in Germany between 2000 and 2019. Furthermore, the number of ADR reports was set in context with the number of inhabitants and the number of assumed drug-exposed inhabitants. In addition, ADR reports informing about off-label use were analysed.

## Material and methods

### Compliance with ethical standards and ethics approval

The study had been approved by the local ethics committee of the Medical Faculty of Bonn (009/17). All ADR reports contained in EudraVigilance are pseudonymised.

### ADRs

An ADR is defined as a response to a medicinal product which is noxious and unintended. The legal definition of an ADR was amended in 2012 [8]. Before 2012 the legal definition was restricted to the use of the drug within the terms of the marketing authorisation. After the amendment the definition included ADRs which have arisen from the use of a medicinal product within and outside the terms of the marketing authorisation such as overdoses, off-label uses, misuses, abuses and medication errors [9]. Our analysis comprises all ADR reports before and after the change of the legal definition of ADRs.

### EudraVigilance

EudraVigilance is the ADR database of the European Medicines Agency (EMA). The EudraVigilance Data Analysis System (EVDAS) is the tool, which is used by the EMA and national competent authorities (NCAs), among others, to monitor the safety of all authorised medicines in the EU [10]. Public access is granted to EudraVigilance although different levels of access apply to different stakeholders [11]. In EudraVigilance, ADRs are coded in accordance with Medical Dictionary for Regulatory Activities (MedDRA) terminology [12].

### Reporting routes of ADRs

Physicians in Germany are obliged by their professional conduct code to report ADRs to their professional councils, the Drug Commission of Pharmacists or to the Drug Commission of the German Medical Association [13] which forward these reports to either the Federal institute for Drugs and Medical Devices (BfArM) or the Paul-Ehrlich-Institute (PEI). The BfArM is responsible for chemically defined drugs, the PEI is responsible for monoclonal antibodies, vaccines etc. [14–16]. The competent authorities forward these ADR reports to EudraVigilance, the ADR database of the European Medicines Agency (EMA). Furthermore HCPs and consumers may also report to the marketing authorization holders which will send these reports to EudraVigilance. Consumers may also report directly to one of the two competent authorities which will then redirect these reports to EudraVigilance [17]. Due to method inherent limitations, analysis in ADR databases like EudraVigilance cannot provide ADR incidences like randomized controlled trials. However, the analysis of ADR reporting, in particular if put in context with external data sources like the number of inhabitants and assumed drug-exposed inhabitants provides other relevant drug safety information and represents an acknowledged pharmacovigilance tool.

### Identification of cases in EudraVigilance

**Fig 1:**
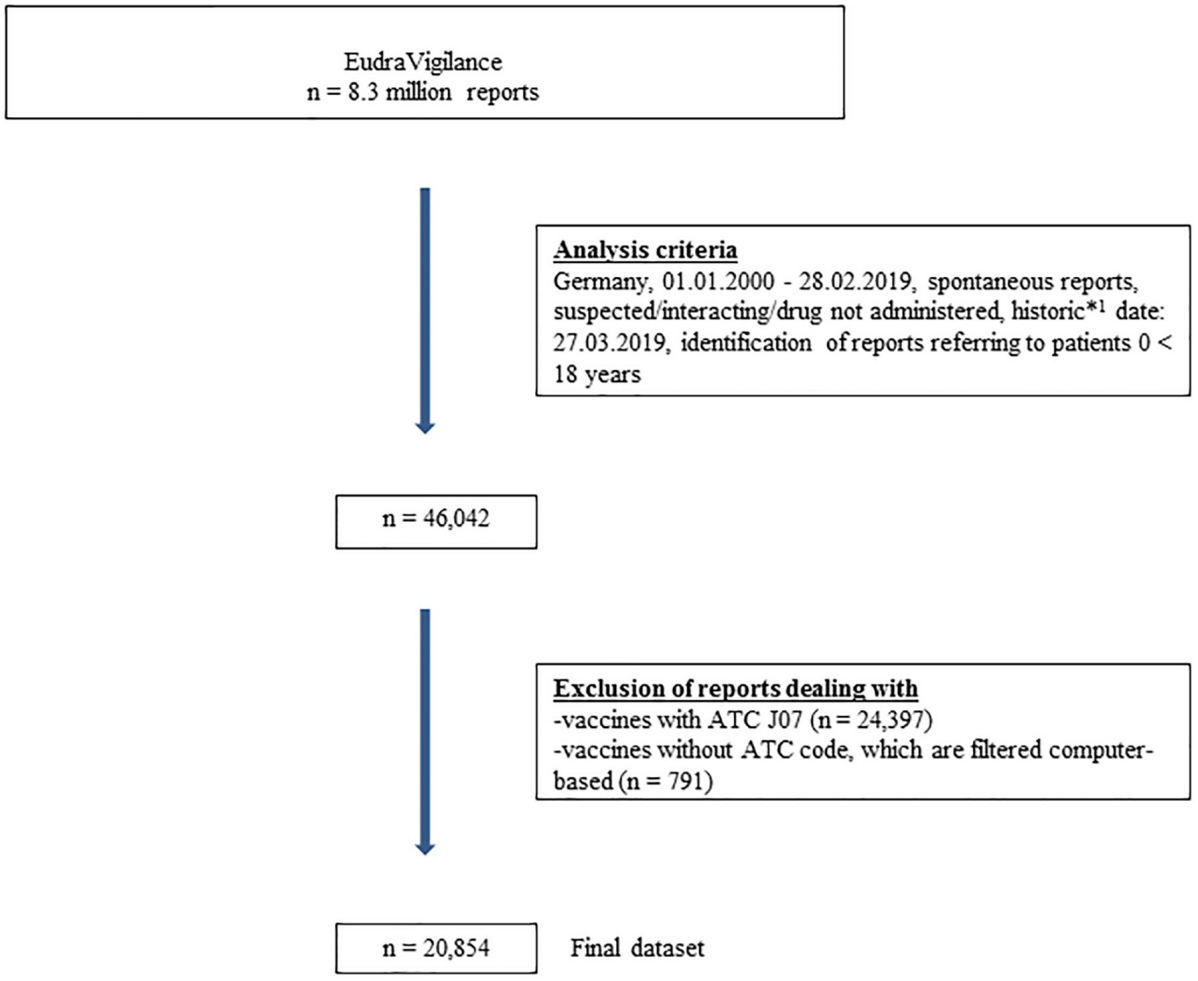
Generation of the final dataset. Fig 1 shows the analysis criteria and the number of ADR reports identified in EudraVigilance with regard to the defined inclusion and exclusion criteria. ^1^The historic date corresponds to the date of the query. This date was set at the beginning and was applied to all subsequent analyses in order to exclude subsequent changes to the dataset (e.g. due to follow-up reports).

All spontaneous ADR reports in which drugs were designated as suspected/interacting originating from Germany and received between 01.01.2000–28.02.2019 were identified in EVDAS (n = 473,344). Therefore, ADR reports collected during an observational study, reports categorised as *study reports* or *other reports*, or those of *unknown origin* (not assigned to any category) were excluded. The dataset was further restricted to reports for children aged 0–17 years (n = 46,042; 9.7%). We also excluded ADR reports referring to vaccines, because vaccination is a medical process which differs from drug administration and is used in specific age intervals, which would affect our age and sex stratified analysis. At the end, the data set consisted of 20,854 spontaneous ADR reports (Fig 1).

### Strategy of analyses

The final dataset was analysed with regard to reporting sources, seriousness criteria, age and sex distribution, time course of annual reports and reports informing about off-label use. Furthermore reporting rates were calculated in context with the number of inhabitants and assumed drug-exposed inhabitants.

### Age and sex distribution

For the age stratified analysis, a modified age stratification according to the National Association of Statutory Health Insurance Physicians was used (“0–1 month”, “2 months—1 year”, “2–3 years”, “4–6 years”, “7–12 years” and “13–17 years”) [18]. These age-stratified groups were analysed by sex.

### Time course of annual reports

The time course of the ADR reports from 2000–2018 was evaluated, especially with regard to the reporting sources (HCP/non-HCP) and the seriousness of the report.

### Seriousness criteria

The classification “serious ADR” corresponds to the legal definition of seriousness in accordance with the GVP guideline [19] and defines any untoward medical occurrence to a medicinal product that results in death, is life-threatening, requires inpatient hospitalisation or prolongation of existing hospitalisation, results in persistent or significant disability or incapacity, or is a congenital anomaly/birth defect [20]. Multiple assignments per ADR report are possible. This definition of seriousness refers to the legal criteria which should not be understood as being identical with the severity of an ADR, which refers to clinical aspects [21].

### Primary reporting source

The primary reporting source defines the person who generated the ADR report. In this respect, a Healthcare Professional (HCP) is a medically-qualified person such as a physician, dentist, pharmacist, nurse, or coroner. In contrast, a non-Healthcare Professional (non-HCP) is defined as a person who is not a HCP such as a patient, lawyer, friend or relative of a patient [9]. The reporting source can be different from the sender of the report, e.g. a marketing authorization holder who forwarded the report to the competent authority. An ADR report may be reported or co-reported by more than one primary source.

In order to analyse if there was an increase in the number of ADR reports only reported by non-HCPs in recent years, the number of ADR reports reported by an HCP was divided by the number of ADR reports from non-HCPs for each year (in brevity “HCP/non-HCP ratio”). Additionally, we calculated the ratio of the number of serious ADR reports divided by the number of non-serious ADR reports (in brevity “serious/non-serious ratio”) independent of the primary reporting source and for HCPs and non-HCPs.

### Number of drug prescriptions in defined daily doses (DDD)

We determined the number of drug prescriptions in defined daily doses (DDD) per insured person for each year from 2000–2018. The number of prescriptions in DDD per insured person per age group was obtained from the annually published prescription data (AVP) [22]. These publicly available drug prescription reports cover all reimbursements for medicinal products prescribed at the expense of the statutory health insurances, excluding over-the-counter drugs and limited to outpatient prescriptions. Hence, drug prescriptions of privately insured patients or inpatients are missing. It is estimated that the drug prescriptions recorded in the drug prescription reports present roughly 90% of the German population [23, 24].

The DDD as a standardized value may deviate from the individually applied or prescribed dose in varying extent depending on the individual drugs [22]. The presented DDD per insured person referred to the age group 0–19 years, which slightly deviated from our target population (0–17 years).

### Reporting rates

The exact number of inhabitants exposed to drugs in Germany is not known. Hence, we set the number of ADR reports in relation to the number of inhabitants and to the number of assumed drug-exposed inhabitants:

1. *Number of ADR reports*: we analysed the number of ADR reports for the age groups 0–2 years, 3–6 years, 7–10 years, 11–13 years and 14–17 years in total and stratified by sex for each of the years 2000–2018.
2. *Number of inhabitants*: we analysed the population figures for the age groups 0–2 years, 3–6 years, 7–10 years, 11–13 years and 14–17 years by sex for each year in the period 2000–2018 using data from the Federal Statistical Office’s GENESIS database (Destatis) [25].
3. *Average number of ADR reports per 100*,*000 inhabitants stratified by age group*: a ratio (i/ii) was calculated for each sex in the respective age group for each year in the period 2000–2018, and then an average value for 2000–2018 was formed. The results for the average number of ADR reports per 100,000 inhabitants are shown in Fig 4A.
4. *Average number of ADR reports per 100*,*000 assumed drug-exposed inhabitants stratified by age group and sex*: the calculation was analogous to iii) except for the number of inhabitants (ii) which was multiplied by the percentage of children and adolescents exposed to drugs according to the KiGGS 1 study from 2007 (Fig 4B) and the KiGGS 2 study from 2019 (Fig 4C). It should be noted that in the KiGGS 2 study [26] the number of drugs administered for the age group 0–2 years was not recorded. In addition, the date of the ADR may differ from the date of the ADR report (e.g. due to delayed reporting). The year of the receive date of the ADR report was used for all ratio calculations, thus, the inaccuracy would apply to all 19 years, reducing any impact [14].

### Off-label use

In order to analyse the number of ADR reports referring to off-label use, we combined appropriate preferred terms (PTs) according to the MedDRA catalogue [12] (S4 Table in S1 File). Possibly, not all ADR reports referring to drugs that were used off-label are coded with one of the aforementioned PTs. Thus, not all ADR reports may have been identified.

### Statistical analysis

Means and medians were calculated for the patients’ age and the increase of ADR reports per inhabitant/assumed drug-exposed inhabitant. We calculated an odds ratio (OR) with Bonferroni confidence interval (CI) adjustment for each age group for males versus females. This was set in relation to the number of males/females in the other age groups, in order to identify differences in sex distribution in the dataset for different age strata.

This dataset has also been subject to a separate investigation which analysed suspected drugs and the reported ADRs (manuscript in preparation).

## Results

### Primary reporting source

The identified 20,854 ADR reports were reported by 23,612 reporting sources. A HCP was involved in reporting the ADR in the majority of cases (86.5%). Only 12.2% were explicitly reported by a non-HCP (Table 1). In 10.3% of those reports in which a HCP was recorded, a non-HCP was noted as additional reporting source. On a more detailed level, 58.4% of the ADR reports were reported by a physician only, 9.4% by a pharmacist only and 12.2% by a consumer only.

**Table 1:** Reported characteristics of the dataset.

	Complete dataset (n = 20,854)
***patients demographics***	
**mean age (median)**	8.5 years (9.0 years)
**Female**	44.9% (n = 9,354)
**Male**	51.2% (n = 10,670)
**Unknown**	4.0% (n = 830)
***seriousness criteria*** ^a^	
**Serious**	74.4% (n = 15,522)
**Death**	2.9% (n = 604)
**life-threatening**	6.6% (n = 1,374)
**hospitalisation**	31.9% (n = 6,655)
**Disabling**	1.7% (n = 358)
**congenital anomaly**	5.5% (n = 1,140)
**Unknown**	4.2% (n = 876)
***primary source*** ^b^	
**HCP**	86.5% (n = 18,036)
**Physician**	58.4% (n = 12,171)
**Pharmacist**	9.4% (n = 1,959)
**non-HCP**	12.2% (n = 2,546)
**Consumer**	12.2% (n = 2,537)
**Unspecified**	1.3% (n = 272)

Table 1 shows an overview of the demographic parameters of the cases identified in EudraVigilance. In addition, seriousness and primary reporting sources for the reported cases were analysed.

^a^ one ADR report may contain information about more than one seriousness criterion, therefore, the number of reported seriousness criteria exceeds the number of ADR reports.

^b^ the term *HCP* summarizes all ADR reports in which a HCP (e.g. pharmacist, physician, other HCP) was involved in reporting the ADR. The term *non-HCP* summarizes all ADR reports in which only a non-HCP (e.g. consumer, lawyer, other non-HCP) was involved in reporting the ADR. Beyond that, Table 1 lists the number of reports which were only reported by physicians, pharmacists or consumers, respectively. The percentage determined refers to the share of the reporting source in the total number of all reports (20,854 reports).

### Seriousness criteria

Over the complete dataset 74.4% of the ADR reports were classified as serious. 2.9% of all ADR reports were marked as fatal. 6.6% of the ADR reports were classified as life-threatening and 31.9% explicitly informed about a hospitalisation or prolongation of an existing hospitalisation (multiple assignments per ADR report were possible) (Table 1). If stratified by age group, the highest number of serious ADR reports was observed for the age group 0–1 month with 93.5% (2,291/2,451). Moreover, within this age group, the highest number of ADR reports relating to the seriousness criterion "congenital anomaly" was found with 39.5% (968/2,451). The highest number of ADR reports designated with the seriousness criterion “death” was seen in the age group 2 months—1 year with 6.1% (141/2,302) (S1 Table in S1 File).

78.7% of the ADR reports originating from physicians were classified as serious and 2.9% as fatal, compared to 53.2% (serious) and 1.7% (fatal) of the consumer reports. Among all seriousness criteria, only the relative share for “congenital anomaly” was higher in consumer compared to physician reports (4.9% versus 4.0%) (S2 Table in S1 File).

### Age and sex distribution

Across the complete dataset male sex slightly dominated. 51.2% of the ADR reports referred to males, 44.9% to females and in 4.0% of the ADR reports the sex was not specified. Male sex was more often reported in every age group between 0–12 years (55.5% males vs. 39.6% females) except for the age group 13–17 years (43.0% males vs. 54.8% females) (S1 Fig in S1 File).

With regard to the age distribution, most of the ADR reports referred to the age group 13–17 years (34.8%), followed by the age groups 7–12 years (25.8%), and 0–1 month (11.8%).

### Time course of annual reports

**Fig 2:**
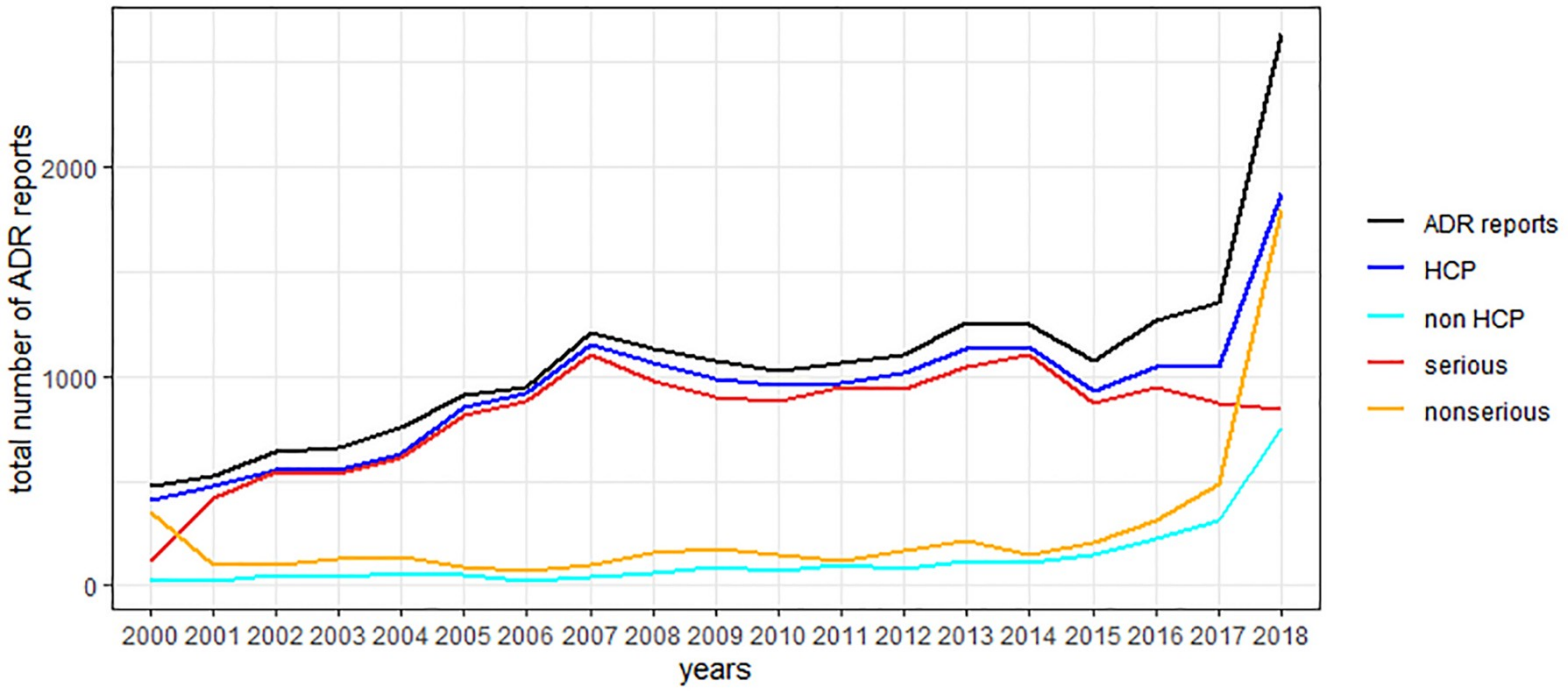
Total number of ADR reports per year stratified by seriousness and primary reporting source.

The total number of reports per year has increased in the time period 01.01.2000–31.12.2018 (Fig 2).

In 1.3% (271/20,854) of the reports the primary reporting source was not specified. Therefore, the number of reports for HCPs and non-HCPs do not exactly add up to the total number of ADR reports. Likewise, in 4.2% of the reports it was unknown if the reaction was serious or non-serious. Hence, the number of serious and non-serious reports does not add up exactly to the total number of ADR reports.

A huge rise in the number of ADR reports was observed in 2018 with almost twice as many ADR reports as for the year 2017. This seems to be caused by a considerable increase of non-serious ADR reports in 2018 (3.7 times compared to 2017). The ratio serious/non-serious ADR reports showed a fluctuation over the years. The lowest ratios of serious/non-serious ADR reports were observed in the years 2000 (ratio: 0.4) and 2018 (ratio: 0.5) whereas the highest ratios were observed in 2006 (ratio: 13) and 2007 (ratio: 10.6). Submission of reports by both, HCPs and non-HCPs increased from 2017 to 2018. However, the relative increase was higher for reports submitted by non-HCPs (factor 2.4) than for reports submitted by HCPs (factor 1.8). Likewise, the ratio of reports submitted by HCPs versus non-HCPs decreased during the time period 2000 (ratio: 21.5) to 2018 (ratio: 2.5) apart from 2006 (ratio: 34.0) and 2007 (ratio: 25.1), where higher ratios were observed.

From 01.01.2000–28.02.2019 the number of reports forwarded by MAHs to EudraVigilance, increased. This can be seen by the relative decrease of reports forwarded by the competent authorities BfArM and PEI to EudraVigilance.

The percentage of reports sent by BfArM/PEI was 85.9% until 31.12.2017 but only 25.7% for 2018.

### Total number of reports per 100,000 inhabitants, assumed drug-exposed inhabitants, and prescription data in the respective years

**Fig 3:**
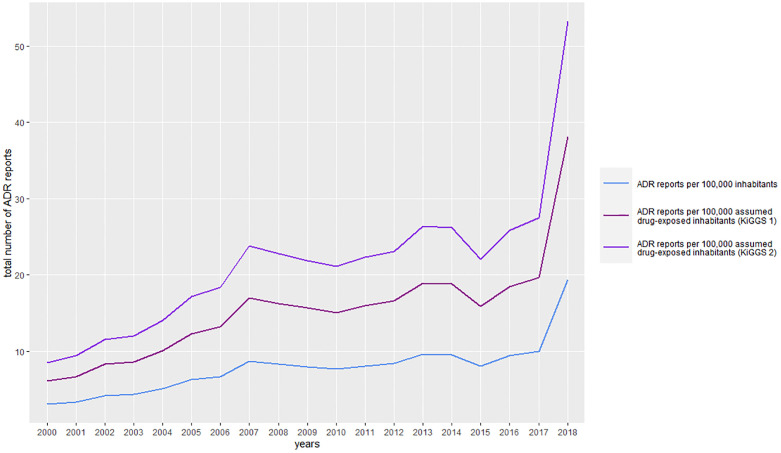
Annual number of ADR reports per 100,000 inhabitants and assumed drug-exposed inhabitants (based on two consecutive published estimations of assumed drug exposure). Fig 3 depicts the annual number of ADR reports per 100,000 inhabitants and assumed drug- exposed inhabitants based on two consecutive published estimations of assumed drug exposure KiGGS 1 [23] and KiGGS 2 [22].

In contrast to the increase of the absolute number of ADR reports from 2000 to 2018, the number of inhabitants aged 0–17 years decreased. (S3 Table in S1 File). The ratio number of *ADR reports/*100,000 inhabitants increased from 2000 (3.1) to 2018 (19.4) (S3 Table in S1 File and Fig 3).

The average ratio over the complete time period was 7.8 [+/- 3.5] ADR reports per 100,000 inhabitants. The average number of *ADR reports/100*,*000 assumed drug-exposed inhabitants* was 15.4 [+/- 7.0], when referring to the drug exposure based on the analysis for the time period 2003–2006 (KiGGS 1) [27]. Referring to the drug exposure based on the analysis for the time period 2014–2017 for KiGGS 2, the average ratio was 21.4 [+/- 9.7] [26]. The number of drug prescriptions in DDD per insured person slightly increased from 610 (2004) to 657 (2018) (S3 Table in S1 File). Interestingly, the DDD per insured person was highest for the age group 0–4 years with 35.5% in relation to the total age group 0–19 years (3,208 DDD/9,046 DDD). Between 2004 and 2018, 30.9–40.7% of all DDDs prescribed for the whole age group 0–19 years referred to this age group.

### Age-stratified analysis of the annual number of reports per 100,000 inhabitants and assumed drug-exposed inhabitants

**Fig 4:**
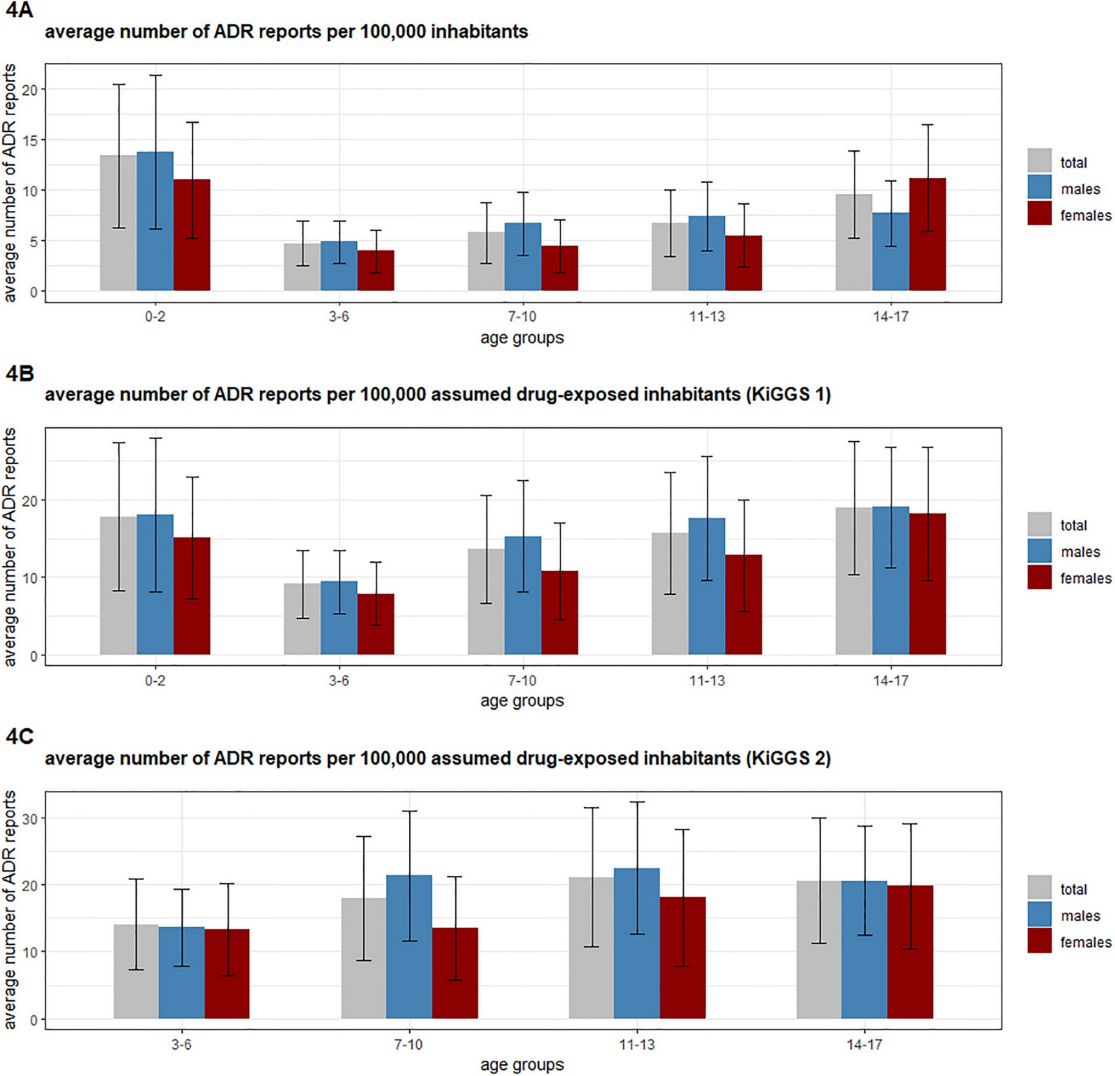
A) Average number of ADR reports per 100,000 inhabitants; B) Average number of ADR reports per 100,000 assumed drug-exposed inhabitants (KiGGS 1 [27]); C) Average number of ADR reports per 100,000 assumed drug-exposed inhabitants (KiGGS 2 [26]). Fig 4A shows the mean (+/- SD) number of ADR reports per 100,000 inhabitants distributed by age and sex. Fig 4B and 4C show the mean (+/- SD) number of ADR reports per 100,000 assumed drug-exposed inhabitants distributed by age and sex. The proportion of drug-exposed children was taken from the KiGGS 1 study (Fig 4B) [27] and the KiGGS 2 study (Fig 4C) [26]. KiGGS 2 [26] does not present the number of applied drugs for the age group 0–2 years, hence, no calculation for this age group is shown in Fig 4C. For the calculation of the assumed drug-exposed inhabitants, the number of inhabitants per year (2000–2018) was multiplied with the proportion of drug-exposed children.

If stratified by age group and covering the complete time period, the highest ratio (iii) (see Material and methods) with approx. 13.4 ADR reports/100,000 inhabitants was calculated for the age group 0–2 years whereas the lowest ratio was observed for the age group 3–6 years (4.7 ADR reports/100,000 inhabitants). Average figures for males were higher than for females in every age group except for the age group 14–17 years (Fig 4A).

If the number of ADR reports was set in relation to the number of 100,000 assumed drug-exposed inhabitants (iv) (in Material and methods) based on data obtained from the KiGGS 1 study [27], the highest ratio was observed for the age class 14–17 years (18.9 ADR reports/100,000 assumed drug-exposed inhabitants), whereas the second highest ratio was calculated for the age group 0–2 years (17.8 ADR reports/100,000 assumed drug-exposed inhabitants).

In contrast, in the KiGGS 2 study the age groups 11–13 years (21.1 ADR reports/100,000 assumed drug-exposed inhabitants) and 14–17 years (20.6 ADR reports/100,000 assumed drug-exposed inhabitants) accounted for the highest ratios. Despite the higher number of ADR reports in absolute terms, in relation to the number of drug-exposed inhabitants, the number of ADR reports for females and males aged 14–17 years was almost equal. When linking the average number of ADR reports/100,000 inhabitants with the exposure data from KiGGS 1, 18.2 ADR reports/100,000 assumed drug-exposed inhabitants were found in females compared with 19.0 ADR reports/100,000 assumed drug-exposed inhabitants in males. By linking the average number of ADR reports/100,000 inhabitants with the exposure data from KiGGS 2, 19.8 ADR reports/100,000 assumed drug-exposed inhabitants were determined for females and 20.6 ADR reports/100,000 assumed drug-exposed inhabitants for males.

### Off-label use

Only 3.5% (722/20,854) of the reports were explicitly designated as off-label (Table 2). 3.7% of these cases were designated as fatal compared to 2.9% of the complete dataset.

**Table 2:** Characteristics of the 722 cases explicitly designated as off-label use.

	Off-label use (3.5%, n = 722)			
^a^ Age groups	total	female	male	unspecified
0–1 month	3.1% (22/722)	1.0% (7/722)	1.5% (11/722)	0.6% (4/722)
2 month—1 year	11.4% (82/722)	4.0% (29/722)	5.5% (40/722)	1.8% (13/722)
2–3 years	7.8% (56/722)	3.1% (22/722)	4.0% (29/722)	0.7% (5/722)
4–6 years	11.9% (86/722)	4.2% (30/722)	6.2% (45/722)	1.5% (11/722)
7–12 years	28.3% (204/722)	11.9% (86/722)	14.1% (102/722)	2.2% (16/722)
13–17 years	37.7% (272/722)	22.4% (162/722)	13.8% (100/722)	1.4% (10/722)
***Seriousness criteria***				
Serious	62.7% (453/722)			
Death	3.7% (27/722)			
Life-threatening	4.8% (35/722)			
Hospitalisation	21.1% (152/722)			
Disabling	1.5% (11/722)			
Congenital anomaly	0.7% (5/722)			

Table 2 shows the number of reports available on off-label use. These reports were classified according to age group and seriousness (without age classification).

^a^ classification into corresponding age groups according to the classification of the National Association of Statutory Health Insurance Physicians in Germany (NASHIP/ KBV) [18].

Most of the reports designated as off-label-use (37.7%; 272/722) referred to the age group of 13–17 years. However, if referring to the number of reports for this age group, only, 3.8% (272/7,251) of the ADR reports related to an off-label use (S4 Table in S1 File). In comparison, the proportion of children aged 4–6 years identified in reports as off-label use was lower with 11.9% (86/722). However, when these reports are related to the total number of reports available for this age group, the highest proportion compared to the other age groups was calculated with 4.5% (86/1,929). In contrast, 3.1% (22/722) of the reports referred to off-label reports related to the age group 0–1 month, while in relation to the respective total number of reports for this age group, only 0.9% (22/2,451) of the ADR reports related to off-label use.

In all age groups there were slightly more reports of off-label use among males than among females, except for the age group 13–17 years (22.4% vs. 13.8%).

## Discussion

To the best of our knowledge, this is the first explicit descriptive analysis of the characteristics and the time course of ADR reports referring to children for Germany in the EudraVigilance database. In addition, this analysis also took external data sources such as exposure and population data into account.

### Age and sex distribution

In our dataset sex was almost equally distributed over the whole dataset with a slight preponderance of males (51.2%) over females (44.9%), as also seen in other studies (28, 29). In contrast, in ADR reports referring *to all ages* in Germany, female sex dominated (54.6% females, 38.8% males) [15]. If the reports were additionally stratified by age, in our analysis male sex was more often reported in patients aged 0–12 years (male 55.5% vs. female 39.6%) and female sex was more often reported in the age group 13–17 years (male 43.0% vs. female 54.8%) as also described in literature [28, 29]. This finding may reflect the higher proportion of females taking any drugs compared to males as observed in a German study [26]. In this respect roughly 21.5% of females aged 14–17 years take hormonal contraceptives [26] and this may contribute to the higher number of drug-exposed females thereby leading to an increase of ADR reports in absolute terms for females aged 14–17 years in Germany. If the number of ADR reports for females is related to the number of assumed drug-exposed inhabitants, the ratios for females and males were almost comparable. Thus, the higher number of ADR reports for females in absolute terms may reflect their higher drug exposure. However, further analyses would be necessary in order to make a more precise statement.

### Seriousness criteria

Over the complete dataset, 74.4% of all ADR reports were classified as serious in accordance with the legal definition [21] (HCP reports: 77.4%; non-HCP reports: 53.4%) (Table 1). In a systematic review of studies of ADRs in children using ADR databases, the percentage of ADRs or reports classified as serious ranged from 2% - 68% [30]. In the analysis in the Food and Drug Administration’s adverse event reporting system (FEARS), which included 36,241 pediatric reports, 62% of them were classified as serious [31]. It is emphasized that the designation of an ADR as serious follows the legal definition which may deviate from the clinical classification of severity of an ADR [21]. In addition, it should be noted that marketing authorization holders have been obliged to report serious ADRs within 15 days, whereas an ongoing obligation to report non-serious ADRs on a regular basis and as individual case reports has been established in 2017 (for a detailed description of the former and actual reporting obligations see [15]). Nevertheless, non-serious reports have also been submitted before. However, a preferential reporting of serious reports could explain to a certain extent the relatively high percentage of cases classified as serious. In total, the percentage of 74.4% from this study is higher than the percentage of 66.9% which has been reported for the complete ADR-dataset for Germany (no age restriction) [15].

For studies performed in the EU with death as an outcome ranged from 0.1% to 2.2% [30] compared to 2.9% in our and 8% in the US study [31]. Differences in the study design (e.g. in- or exclusion of vaccine reports) may account among others for this difference.

### Primary reporting source

In our analysis 86.5% of all reports were reported or co-reported by HCPs and 12.2% of the reports were from non-HCPs, such as consumers and lawyers (reports from lawyers < 1%). These figures seem to be roughly in line with data from other studies which, however, reported separate figures for physicians, pharmacists and other HCPs like nurses [29, 32–34].

The ratio *serious vs*. *non-serious ADR reports* was higher for HCP reports (4.2: 1) than forth non-HCP reports (1.2: 1). This suggests that non-HCPs tend to report more frequently non-serious ADRs as compared to HCPs. In an analysis of ADR reports for children from European consumers, about 60% were designated as serious [35] which compares to 53.4% in our analysis. However, since the far majority of reports in our analysis originated from HCPs (n = 18,036; 86.5%), their share among the non-serious reports clearly outweighs the non-serious reports from non-HCPs.

### Total number of reports per 100,000 inhabitants, assumed drug-exposed inhabitants, and prescription data in the respective years

As already observed in a previous analysis of ADR reports referring to patients of all ages, we also observed a continuous increase in the annual number of ADR reports for children and adolescents from 2000–2018 [15]. However, the significance of the absolute number of ADR reports per year as a stand-alone figure is limited [36]. Hence, we set it in relation to the number of inhabitants, assumed drug-exposed inhabitants, and publicly available prescription data in the respective years (see further below).

Over the complete period our calculated average number of ADR reports/100,000 inhabitants was 7.8. This compares well to a systematic review of studies of ADRs in children using ADR databases which found ADR reporting rates for studies originating from the EU from 1.1 to 23.8 ADR reports/100,000 inhabitants [30].

We also analysed the course of the annual number of ADR reports in relation to the number of inhabitants per year in the study period. Conversely to the increase of ADR reports from 2000–2018 (2000: 477 reports/year; 2018: 2,633 reports/year), the number of children declined (2000: 15.5 million children; 2018: 13.6 million children). Therefore, the observed increase in reports is not due to an increase of children in this time period. In contrast, the proportion of children exposed to drugs has decreased from the KiGGS 1 study (46.4%) to the KiGGS 2 study (36.4%) (this finding may also be due to differences in the study designs of the two epidemiological studies).

As a next step we calculated the number of assumed drug-exposed inhabitants as a denominator using data from the two German KiGGS studies [26, 27]. These ratios also increased over the analysed time period (Fig 4B and 4C).

We also investigated the annual drug exposure in terms of defined daily doses (DDD) [37] per insured person aged 0–19 years. An increase in DDDs per insured person aged 0–19 years was observed in the prescription reports between 2000 and 2018 (S3 Table in S1 File). However, it cannot be differentiated whether this increase is due to more children being exposed to drugs or to more children being exposed to more than one drug. Nevertheless, both scenarios would probably increase the incidence of ADRs and could therefore contribute to an unknown extent to the increase in the number of ADR reports per year.

In summary, the different approaches to combine the number of ADR reports with other sources (inhabitants or exposure data) pointed into the same direction, suggesting that the increase in the total number of ADR reports in the time period 01.01.2000–28.02.2019 is not essentially due to an increase of the pediatric population. Instead other factors may apply which are discussed below.

First, as discussed previously, changes of the reporting obligations, in particular following the release of the new pharmacovigilance legislation in 2012 [8], could account to some extent for the increase. As one consequence, the definition of the term ADR was widened and now includes also those ADRs which occurred with the use of the drug outside its authorised conditions.

Second, since 2017 all non-serious ADRs have to be reported as a unique/single ADR report electronically within 90 days to the EudraVigilance database in accordance with the legal requirements [8]. This may partially explain the sharp increase in non-serious ADR reports in 2018 (2017: 484 non-serious ADR reports; 2018: 1,793 non-serious ADR reports; + 370%).

Third, the number of non-HCP reports has steadily increased from 19 reports in 2000 with a peak share of 759 reports in 2018 (+ 3,995%). The aforementioned new pharmacovigilance legislation obliged all EU member states to establish patient/consumer reporting within their spontaneous reporting systems [8]. Although consumer reports have been regularly published in Germany, their share has increased significantly in recent years [15].

Fourth, in 2007 the Pediatric Regulation was implemented in the EU [38] and a new guidance on reporting ADRs in children was published in Germany [39]. Both measures may have stimulated reporting from physicians and the pharmaceutical industry [39]. In fact, from 2006 to 2007, the number of reports rose from 952 to 1210.

Fifth, the increase in ADR reports could also reflect a generally increased consciousness for ADRs by HCPs and consumers and the motivation to report them, facilitated by an easier access to information in the internet and the possibility to report ADRs online [15].

All these factors may have contributed to a different extent to an increase of the ADR reports in the time period investigated. However, it is not possible to define the individual contribution of each single factor.

### Age stratified analysis of the annual number of reports per 100,000 inhabitants and assumed drug-exposed inhabitants

The age group 3–6 years has the lowest ratio of ADR reports/inhabitant (Fig 4A) and assumed drug-exposed inhabitant (Fig 4B and 4C). In contrast, the highest ratios were observed for the age groups 0–2 years and 14–17 years. It should be noted that the age group 0–2 years includes neonates (0 days—1 month), to which 11.8% of all ADR reports referred to. Exposure in this age group may be due to the mother taking medication during pregnancy. In addition, one may speculate that children aged 0–2 years receive more often drugs, thereby increasing the likelihood of ADRs. In fact, the first KiGGS study reported that roughly 74.9% of the age group 0–2 years took any medication [27]. This huge percentage may be due to the youngest patients (0–6 month) for which the prevalence of drug intake was estimated to be as high as 92%. In addition, there are three recommended and reimbursed presentations to pediatric physicians in Germany in the time period 0–2 months [40]. These presentations to pediatric physicians may increase the likelihood of ADRs being detected and reported. For instance malformation if suspected to be caused by a drug may be more likely reported in the first months after birth than later. In our analysis, there were 968 reports of malformations among the 2,451 reports for the age group 0–1 month to 2 years. There may also be a higher risk of ADRs in this age group due to the special pharmacokinetics and pharmacodynamics [41] which may impact on the tolerance of drug therapy. However, in clinical practice this should be taken into account by the prescribing physician. In contrast, children aged 14 to 17 years had a slightly higher ratio of ADR reports/assumed drug-exposed inhabitant (Fig 4B) although their drug exposure was estimated to be lower than for the age group 0–2 years in the first KiGGS study (50.7% vs. 74.9%) [27]. Differences in drug prescriptions for these two age groups could account for this finding. The youngest age group may be more often treated with drugs possessing a lower intrinsic potential to cause ADRs, like supplements (e.g. fluoride) compared to the age group 14–17 years in which for instance psychotropic drugs are administered. In addition, there are also only two recommended and reimbursed presentations to pediatrics for this age group (between 12–14 and 16–17 years) [40].

### Off-label use

Many medicinal products are used off-label in children, i.e. outside their approved indication or other approval conditions [42]. For Germany, it was found that about 30% of all attributable medications have been used off-label in pediatrics [43]. One important reason for the extensive off-label use in children could be a lack of appropriate clinical trials in this population and a lack of age-appropriate formulations [44, 45]. Therefore, the analysis of spontaneous ADR reports can be an important tool to gain further knowledge on the safety of drugs used in children, especially those used off-label [15].

Across our complete dataset only 3.5% (722/20,854) of the ADR reports were explicitly designated with an off-label use. This figure is considerably lower than the proportion identified in the context of a post-marketing pharmacovigilance program, in which 8% of ADR reports were identified with an off-label use [46]. In other ADR database analyses, the percentages for off-label use were much higher than in our analysis, ranging from 17% (EU; database analysis) [35] to 42% (Sweden; single case analysis) [47]. In a recent systematic review covering worldwide studies on drug prescriptions from 1994–2013, off-label prescriptions ranging from 12.2% to 70.6% were reported [48].

The figure calculated in our analysis appears rather low in view of a reported prevalence rate in Germany of self-reported off-label use of 40.2% [43]. Missing explicit designation of an ADR report as “off-label” may account to some extent for the lower numbers of ADR reports referring to off-label use in our analyses. If the reporter did not explicitly indicate the off-label use in the ADR report, it would not be designated as off-label in accordance with the MedDRA terminology in the ADR-database, even if it truly represents an off-label use. The reason for not explicitly indicating the off-label use may vary, including ignorance and fear of legal consequences when reporting an ADR in association with a drug used off-label to the professional council of the national competent authority.

In addition, the legal definition of an ADR was widened in 2012 [8], since then including also those ADRs which occurred with the use of the drug outside authorised conditions, i.e. off-label use. Although ADR reports describing off-label use have been received before 2012 the number of these reports may have increased thereafter. Our analysis was based on ADR reports received between 01.01.2000–28.02.2019, hence, this legal aspect could also have contributed to some extent to our finding of only 3.5% of reports being explicitly designated as off-label.

### Advantages and disadvantages of analysis using spontaneous reporting data

Strengths of our analysis include the large number of ADR reports included in the analysis (n = 20,854 ADR reports) which originated over a long period from 01.01.2000–28.02.2019. In addition, the ADR reports were set in context with inhabitants and aggregated exposure data based on two different sources, epidemiological surveys [26, 27] and reimbursed prescriptions [37]. With regard to the exposure data in terms of reimbursed prescriptions, it is pointed out that this data refers only to members of the statutory health insurance (about 90% of the German population) and does not contain in-patient drug exposure and OTC use [23, 24]. In contrast, the ADR reports do not share these restrictions, however, other method inherent limitations apply to them [36]. One important point among these is underreporting, which may differ, among others, per age group [36, 49]. Underreporting as well as stimulated/preferential reporting and their extent may depend on the ADR itself (e.g. known/unknown, clinical severity), the reported drug (e.g. known for decades vs. recently introduced to the market (so called Weber effect [50]) or the reporting source (e.g. HCPs vs. non-HCPs). In addition, media attention or changed legal requirements may impact on the reporting of particular ADRs or drugs. However, it is impossible to determine the individual contribution of each single effect on the number of ADR reports.

## Conclusion

The continuous increase in ADR reports since 2000 in conjunction with the high proportion of serious ADR reports underlines the importance of drug safety in this vulnerable population. In particular the age group 0–1 years deserves special attention with respect to the high percentage of serious ADR reports (93.5%). HCPs and non-HCPs should be encouraged and trained to report ADRs resulting from off-label use.

## Supporting information

S1 File (DOCX)
