# Supplementary material for: Analysis of the reporting of adverse drug reactions in children and adolescents in Germany in the time period from 2000 to 2019
Source: PLoS One. 2021 Mar 3;16(3):e0247446. doi: 10.1371/journal.pone.0247446 (PMC7928460; doi:10.1371/journal.pone.0247446)
Supplement: S1 File — (DOCX) [file pone.0247446.s001.docx]

**S1 Table. Stratification of seriousness criteria of the ADR reports per age group.**

| age groups | 0-1 month  (n = 2,451) | 2 month-1 year  (n = 2,302) | 2-3 years  (n = 1,537) | 4-6 years  (n = 1,929) | 7-12 years  (n = 5,384) | 13-17 years  (n = 7,251) |
| --- | --- | --- | --- | --- | --- | --- |
| seriousness criteria | | |  |  |  |  |
| serious  death  life-threatening  hospitalization  disabling  congenital anomaly | 93.5 % (n = 2,291)  4.8 % (n = 118)  6.4 % (n = 158)  21.8 % (n = 535)  2.4 % (n = 58)  39.5 % (n = 968) | 77.5 % (n = 1,784)  6.1 % (n = 141)  7.0 % (n = 161)  39.4 % (n = 908)  1.8 % (n = 41)  6.6 % (n = 152) | 66.8 % (n = 1,027)  2.1 % (n = 32)  4.6 % (n = 70)  32.2 % (n = 497)  0.9 % (n = 14)  0.8 % (n = 12) | 65.9 % (n = 1,272)  3.4 % (n = 66)  5.1 % (n = 99)  27.2 % (n = 524)  1.5 % (n = 29)  0.2 % (n = 4) | 68.4 % (n = 3,682)  1.8 % (n = 97)  6.8 % (n = 365)  29.6 % (n = 1,595)  1.8 % (n = 97)  0.0 % (n = 2) | 75.4 % (n = 5,466)  2.1 % (n = 150)  7.2 % (n = 521)  35.8 % (n = 2,596)  1.6 % (n = 119)  0.0 % (n = 2) |

S1 Table shows an overview of the 20,854 reports identified, classified by age group. The reports that were available for the respective age groups were further analysed with regard to the seriousness criteria. The sum of the individual reports on the respective seriousness criteria may exceed the sum of the reports per age group, as multiple responses to the seriousness criteria per report were possible.

**S2 Table. Stratified analysis of the ADR reports submitted by physicians and consumers with regard to the seriousness criteria.**

|  | physician (n = 12,171) | consumer (n = 2,537) |
| --- | --- | --- |
| *seriousness criteria* | |  |
| serious  death  life-threatening  hospitalisation  disabling  congenital anomaly | 78.7 % (n = 9,578)  2.9 % (n = 350)  8.5 % (n = 1,029)  38.0 % (n = 4,626)  1.8 % (n = 225)  4.0 % (n = 488) | 53.2 % (n = 1,350)  1.7 % (n = 43)  2.2 % (n = 56)  20.6 % (n = 522)  1.1 % (n = 29)  4.9 % (n = 124) |

S2 Table shows an overview in which the reporting sources *physician* and *consumer* were compared. 58.4 % (12,171/20,854) of the reports were reported by physicians, 12.2 % (2537/20854) of the reports were reported by consumers. The corresponding reports per reporting source were further differentiated into the reported seriousness criteria.

| **Year** | **2000** | **2001** | **2002** | **2003** | **2004** | **2005** | **2006** | **2007** | **2008** | **2009** | **2010** | **2011** | **2012** | **2013** | **2014** | **2015** | **2016** | **2017** | **2018** |  |
| --- | --- | --- | --- | --- | --- | --- | --- | --- | --- | --- | --- | --- | --- | --- | --- | --- | --- | --- | --- | --- |
| Number of ADR reports | 477 | 525 | 645 | 656 | 758 | 910 | 952 | 1,210 | 1,135 | 1,077 | 1,026 | 1,068 | 1,103 | 1,256 | 1,251 | 1,074 | 1,265 | 1,354 | 2,633 |  |
| Data from the Federal Statistical Office (1) | | | | | | | | | | | | | | | | | | | | |
| Number of German inhabitants 0-17 years in Mio | 15.5 | 15.4 | 15.2 | 15.1 | 14.8 | 14.6 | 14.2 | 14.0 | 13.7 | 13.5 | 13.3 | 13.1 | 13.1 | 13.1 | 13.1 | 13.3 | 13.5 | 13.5 | 13.6 |  |
| Ratio number of ADR reports per 100,000 inhabitants | 3.1 | 3.4 | 4.2 | 4.3 | 5.1 | 6.2 | 6.7 | 8.6 | 8.3 | 8.0 | 7.7 | 8.2 | 8.4 | 9.6 | 9.6 | 8.1 | 9.4 | 10.0 | 19.4 |  |
| Data from the drug prescription reports (2) | | | | | | | | | | | | | | | | | | | | |
| Sum of the number of drug prescriptions in defined daily doses (DDD) per insured person 0-19 years | - | - | - | - | 610 | 617 | 550 | 573 | 562 | 545 | 543 | 519 | 655 | 661 | 648 | 642 | 639 | 625 | 657 |  |
| Number of drug prescriptions in defined daily doses (DDD) per insured person 0-4 years | - | - | - | - | 209 | 230 | 211 | 218 | 227 | 222 | 217 | 204 | 215 | 214 | 209 | 212 | 210 | 207 | 203 |  |
| Number of drug prescriptions in defined daily doses (DDD) per insured person 5-9 years | - | - | - | - | 146 | 138 | 112 | 119 | 106 | 99 | 95 | 92 | 140 | 142 | 135 | 130 | 133 | 129 | 125 |  |
| Number of drug prescriptions in defined daily doses (DDD) per insured person 10-14 years | - | - | - | - | 108 | 107 | 87 | 90 | 81 | 76 | 76 | 76 | 131 | 135 | 131 | 129 | 129 | 127 | 122 |  |
| Number of drug prescriptions in defined daily doses (DDD) per insured person 15-19 years | - | - | - | - | 147 | 142 | 140 | 146 | 148 | 148 | 155 | 147 | 169 | 170 | 173 | 171 | 167 | 162 | 206 |  |

**S3 Table. Annual number of ADR reports, number of inhabitants aged 0-17 years, number of drug prescriptions in DDD per insured person.**

S3 Table shows that the number of reports per year, the ratio *number of ADR reports per 100,000 inhabitants* and, for the most part, the DDD per age group are increasing. The only decrease is in the population of 0-17 year olds in Germany.

**S4 Table. Reports on ^1^off-label use stratified per age group based on the total dataset.**

|  | **Stratification of the reports on off-label use (3.5 %, n= 722) per age and sex based on 20,854 reports (total dataset)** | | | |
| --- | --- | --- | --- | --- |
| Age groups (total number of reports per age group in the total dataset) | total | female | male | unspecified |
| 0 - 1 month (2,451) | 0.9 % (22/2,451) | 0.7 % (7/986) | 0.8 % (11/1,329) | 2.9% (4/136) |
| 2 month - 1 year (2,302) | 3.6 % (82/2,302) | 3.2 % (29/898) | 3.3 % (40/1,212) | 6.8 % (13/192) |
| 2 - 3 years (1,537) | 3.6 % (56/1,537) | 3.3 % (22/669) | 3.7 % (29/775) | 5.4 % (5/93) |
| 4 -6 years (1,929) | 4.5 % (86/1,929) | 3.8 % (30/787) | 4.3 % (45/1,049) | 11.8 % (11/93) |
| 7 - 12 years (5,384) | 3.8 % (204/5,384) | 4.2 % (86/2,042) | 3.2 % (102/3,186) | 10.3 % (16/156) |
| 13 - 17 years (7,251) | 3.8 % (272/7,251) | 4.1 % (162/3,972) | 3.2 % (100/3,119) | 6.3 % (10/160) |

S4 Table shows the distribution of the 722 reports of off-label use within the 20,854 reports from the total dataset to the corresponding age groups, differentiated by sex.

^1^In order to analyze the number of ADR reports referring to off-label use, we combined appropriate preferred terms (PTs) according to the MedDRA catalogue (3). Included were the PTs “drug effective for unapproved indication”, “drug ineffective for unapproved indication”, “product use in unapproved indication”, “unintentional use for unapproved indication”, “off-label-use”, “product use in unapproved therapeutic environment”, “therapeutic product effective for unapproved indication”, “therapeutic product ineffective for unapproved indication”, “device use issue”, “product use issue”, “intentional device use issue”, “intentional product use issue” and “off-label-use of device”.

**S1 Fig. Number of ADR reports stratified by age group and sex.**

**
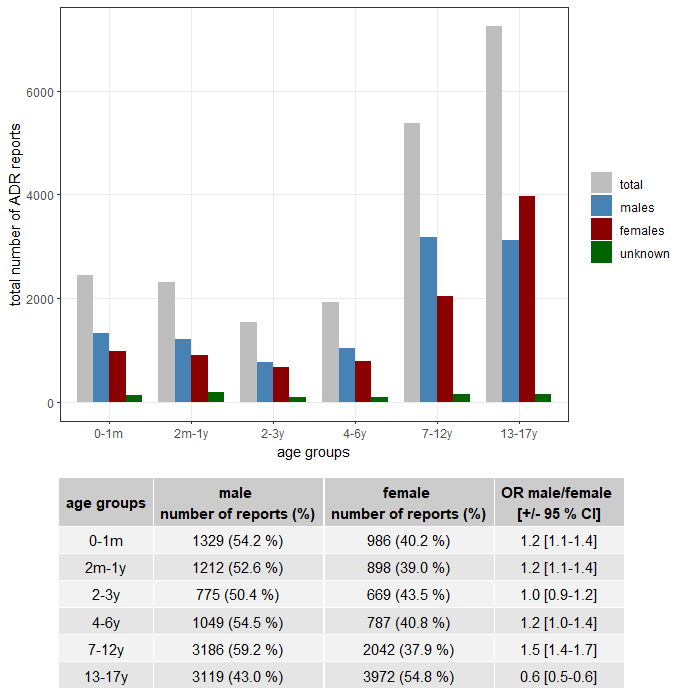
**

This figure shows the number of ADR reports stratified by age groups according to the classification of the National Association of Statutory Health Insurance Physicians in Germany (NASHIP/ KBV) (4) and by sex. Please note that the defined age groups represent age strata of different sizes. In addition, the odds ratio for males/females within each age group was calculated with respect to the other age groups (Bonferroni confidence interval adjustment).

References

1. Statistisches Bundesamt. GENESIS - Die Datenbank des Statistischen Bundesamtes 2020 [Available from: <https://www-genesis.destatis.de/genesis/online>.

2. Wissenschaftliches Institut der AOK (WIdO). Arzneiverordnungs-Report 2000-2019 [Available from: <https://www.wido.de/publikationen-produkte/buchreihen/arzneiverordnungs-report/>.

3. Medical Dictionary for Regulatory Activities. MedDRA 2020 [Available from: <https://www.meddra.org/>.

4. Kassenärztliche Bundesvereinigung. 4.3.5 Altersgruppen 2020 [Available from: <https://www.kbv.de/tools/ebm/html/4.3.5_162395004446927562274884.html>.
